# *In Vitro* Cytotoxic, Antioxidant, and Antimicrobial Activities of *Mesua beccariana* (Baill.) Kosterm., *Mesua ferrea* Linn., and *Mesua congestiflora* Extracts

**DOI:** 10.1155/2013/517072

**Published:** 2013-09-08

**Authors:** Soek Sin Teh, Gwendoline Cheng Lian Ee, Siau Hui Mah, Yoke Keong Yong, Yang Mooi Lim, Mawardi Rahmani, Zuraini Ahmad

**Affiliations:** ^1^Department of Chemistry, Faculty of Science, Universiti Putra Malaysia, 43400 Serdang, Selangor, Malaysia; ^2^School of Biosciences, Taylors University, Lakeside Campus, 47500 Subang Jaya, Selangor, Malaysia; ^3^Department of Human Anatomy, Faculty of Medicine and Health Sciences, Universiti Putra Malaysia, 43400 Serdang, Selangor, Malaysia; ^4^Faculty of Medicine and Health Science, Universiti Tunku Abdul Rahman, 43000 Kajang, Selangor, Malaysia; ^5^Department of Biomedical Science, Faculty of Medicine and Health Sciences, Universiti Putra Malaysia, 43400 Serdang, Selangor, Malaysia

## Abstract

The *in vitro* cytotoxicity tests on the extracts of *Mesua beccariana*, *M. ferrea*, and *M. congestiflora* against Raji, SNU-1, HeLa, LS-174T, NCI-H23, SK-MEL-28, Hep-G2, IMR-32, and K562 were achieved using MTT assay. The methanol extracts of *Mesua beccariana* showed its potency towards the proliferation of B-lymphoma cell (Raji). In addition, only the nonpolar to semipolar extracts (hexane to ethyl acetate) of the three *Mesua* species indicated cytotoxic effects on the tested panel of human cancer cell lines. Antioxidant assays were evaluated using DPPH scavenging radical assay and Folin-Ciocalteu method. The methanol extracts of *M. beccariana* and *M. ferrea* showed high antioxidant activities with low EC_50_ values of 12.70 and 9.77 **μ**g/mL, respectively, which are comparable to that of ascorbic acid (EC_50_ = 5.62 **μ**g/mL). Antibacterial tests were carried out using four Gram positive and four Gram negative bacteria on *Mesua beccariana* extracts. All the extracts showed negative results in the inhibition of Gram negative bacteria. Nevertheless, methanol extracts showed some activities against Gram positive bacteria which are *Bacillus cereus*, methicillin-sensitive *Staphylococcus aureus* (MSSA), and methicillin-resistant *Staphylococcus aureus* (MRSA), while the hexane extract also contributed some activities towards *Bacillus cereus*.

## 1. Introduction

Traditional medicines for human diseases have been widely used in many parts of the world. Herbal plants are usually the primary source of medicine in many developing countries. Natural product compounds from plants provide biologically active compounds, many of which have been developed as new lead chemicals for pharmaceuticals [1]. Only a limited number of plants have been scientifically explored from the many plant species worldwide. Cancer which is the uncontrolled growth of abnormal cells in the body [2] is a serious health problem. Treatments include surgery, chemotherapy, and radiation therapy. Many cancers have developed resistance to prolonged chemotherapy. Hence, there is a need to develop more effective and safer medicines such as herbal medicines. Free radical, a dangerous threat worldwide, causes aging and attacks and damages our immune systems which can lead to chronic inflammation of human cells and body. Recent reports revealed that free radicals provoke the neurodeterioration disorder of human beings which can lead to diseases such as Alzheimers [3] and Parkinsons [4, 5] diseases. Therefore, antioxidants are required to reduce and prevent oxidative damage. *Mesua* species belong to the Clusiaceae family and consist of many health-promoting bioactive compounds [6–10]. Recent pharmacognosy analysis on *Mesua ferrea* leaf and fruit extracts showed both methanolic extracts to exhibit good antibacterial activity towards *Staphylococcus aureus* [11]. Previous research papers on *Mesua* species have shown that these plants exhibit many biological activities such as antioxidant [12–14], hepatoprotective [12], antiarthritic [15], immunomodulatory [16], antibacterial [17], antiacetylcholinesterase (AChE) [18], and larvicidal [19] activities, as well as antiproliferative effects [9, 20–22]. These plants were thus selected for our pharmacological investigation. The ultimate aim of this study was to discover plants with promising bioactivities which can then be developed into drugs through preclinical and clinical developments. Our ongoing research is focused on the screening of the extracts of *Mesua beccariana*, *Mesua ferrea*, and *Mesua congestiflora* against a panel of human cancer cell lines and DPPH scavenging agent. Furthermore, antimicrobial assay was also carried out using four Gram positive and four Gram negative bacteria on the *Mesua beccariana* extracts. Preliminary screening results of cytotoxic and antioxidant activities for *Mesua beccariana*, *Mesua ferrea,* and *Mesua congestiflora* will be reported. We have reported a novel cyclodione coumarin together with other known compounds from *Mesua beccariana* in a previous paper [23].

## 2. Materials and Methods

### 2.1. Plant Material

The stem bark of *Mesua beccariana* (Baill.) Kosterm., root bark of *Mesua ferrea* Linn., and roots of *Mesua congestiflora* were collected from the Sri Aman district in Sarawak, Malaysia. All the plant materials were identified by Associate Professor Dr. Rusea Go, Department of Biology, Faculty of Science, Universiti Putra Malaysia and deposited in the herbarium of the Department of Chemistry, Faculty of Science, Universiti Putra Malaysia. The voucher specimen numbers for *M. beccariana*, *M. ferrea*, and *M. congestiflora* are RG211, RG2694, and RG203, respectively.

### 2.2. General

All the apparatus used for cell culture were sterilized and decontaminated using Hirayama HICLAVE HVE-50. Cell culture handling was carried out in an ESCO Class II BSC Biosafety Cabinet. The healthy cells were spun down, adhered together, and separated from unhealthy and dead cells by using Thermo Scientific Sorvall ST 16R centrifuge machine. All cultures were incubated in 5% CO_2_ humidified incubator at 37°C (ESCO Celculture CO_2_ Incubator with model number CCL-170B-8). Cell stocks were placed in a −86°C  ultralow temperature freezer (Scancool SCL 50 P) and preserved in a liquid nitrogen tank (Taylor-Wharton LS300).

### 2.3. Preparation of Plant Extraction

The aerial parts of the plant samples were extracted using the conventional extraction method in which solvent was added and removed in batches. The dried and milled samples were soaked in nonpolar (hexane), medium polar (dichloromethane, or ethyl acetate) and polar (methanol) solvents in a polarity increasing order. The solvent was decanted and replaced with new solvent after 48 hours.

The air-dried and powdered *Mesua beccariana* stem bark, *Mesua ferrea* roots, and *Mesua congestiflora* stem bark were extracted successively with n-hexane (Hex), dichloromethane (DCM), ethyl acetate (EA), and methanol (MeOH). The extracts were dried under reduced pressure using a rotary evaporator.  Table 5 shows the weights of the extracts obtained from the three *Mesua* species.

### 2.4. Cytotoxicity Assay (MTT Assay)

Preliminary screening tests for cytotoxic activity against a panel of human cancer cell lines on the crude extracts and quercetin (standard) were undertaken by using MTT (3-(4,5-dimethylthiazol-2-yl)-2,5-diphenyltetra zolium bromide) assay. The nine tested cancer cell lines were Raji (human B lymphocyte), SNU-1 (human gastric carcinoma), K562 (human erythroleukemia cells), LS-174T (human colorectal adenocarcinoma), HeLa (human cervical cells), SK-MEL-28 (human malignant melanoma cells), NCI-H23 (human lung adenocarcinoma), IMR-32 (human neuroblastoma), and Hep-G2 (human hepatocellular liver carcinoma). The Raji, SNU-1, K562, HeLa, Hep-G2, and NCI-H23 cells were maintained in RPMI-1640 supplemented with 10% fetal bovine serum (FBS), while the LS-174T, SK-MEL-28, and IMR-32 were maintained in MEM supplemented with 10% FBS. All the cell lines were cultured in 75 cm^2^ T-flask and maintained at 37°C in 5% CO_2_ humidified incubator. The MTT assay is based on the protocol illustrated by Mosmann [24] and was executed in 96-well flat bottom plates. The varying concentrations of 3.13, 6.25, 12.50, 25.00, 50.00, and 100.00 *μ*g/mL were prepared by a serial dilution method. An aliquot of 100 *μ*L of each substock with different concentrations was added to each well together with 100 *μ*L of selected cells to give concentrations of 100.00, 50.00, 25.00, 12.50, 6.25, and 3.13 *μ*g/mL and made up to a final volume of 200 *μ*L in each well. 200 *μ*L of cells with no extracts (untreated cell control—positive control) and 200 *μ*L of medium only (blank medium—negative control) were prepared in the same plate. Each of the samples and controls were prepared in triplicates.

 The plate was then incubated for 72 hours at 37°C in 5% CO_2_ humidifier incubator. After 72 hours, 20 *μ*L of MTT solution was added to all the wells and incubated for 3 hours in a 5% CO_2_ humidifier incubator. The plate was then spun at 3000 rpm for 10 minutes. 160 *μ*L of supernatant from each well was discarded and then added with 160 *μ*L of DMSO to dissolve the purple formazan crystals.

The absorbance of each well was determined using a microplate reader at 550 nm. The average absorbance of each crude extract was calculated, and the average value was used to determine the percentage of cell viability by using the following formula:

(1) Percentage  of  cell  viability =A(average)−B(average)C(average)−B(average)×100,

 where *A* is the absorbance of sample, *B* is the absorbance of negative control, and *C* is the absorbance of positive control.

A graph of percentage of cell viability versus concentration was plotted for each extract. The half maximal inhibitory concentration (IC_50_) values were obtained from the plotted graph. Further dilutions will only be performed on the extracts with IC_50_ values less than 3.13 **μ**g/mL. Three independent experiments were conducted to assure the accuracy of the results. Quercetin was used as standard drug throughout the cytotoxicity experiments.

### 2.5. Antioxidant Assay

#### 2.5.1. DPPH Scavenging Assay

The DPPH (2,2-diphenyl-1-picrylhydrazyl) radical scavenging assay was used to evaluate the antioxidant property of the sample. The assay was performed in 96-well flat bottom plates. The DPPH solution was prepared in a stock solution of 5 mg/2 mL in ethanol (EtOH) and wrapped with aluminium foil. The varying concentrations of 3.13, 6.25, 12.50, 25.00, 50.00, and 100.00 **μ**g/mL were prepared by serial dilution method. An aliquot of 200 **μ**L of each substock with different concentrations was added to each well together with 20 **μ**L of DPPH solution. 200 **μ**L of EtOH and 20 **μ**L of DPPH solution (blank medium—negative control) were prepared in the same plate. Each sample and the control were prepared in triplicates. The plate was then incubated in a dark room at room temperature for 30 minutes. The absorbance of each well was recorded after 30 minutes using a microplate reader at 517 nm. The average absorbance of each crude extract was calculated, and the average value was used to determine the percentage of total radical scavenging activity by using the following formula:

(2) Percentage  of  total  radical  scavenging  activity =A(average)−B(average)A(average)×100,

where, *A* is the absorbance of blank, and *B* is the absorbance of sample.

A graph of percentage of total radical scavenging activity versus concentration was plotted for each extracts. The half maximal effective concentration (EC_50_) values were obtained from the plotted graphs. Three independent experiments were conducted to ensure the precision of the results. Ascorbic acid (vitamin C) was used as the standard drug.

#### 2.5.2. Total Phenolic Content (TPC)

The TPC analysis is to analyze the total phenolic content present in the extract. The TPC analyses were carried out in 24-well flat bottom plates. The extract was initially oxidized by Folic-Ciocalteu reagent (FC) and subsequently neutralized by sodium bicarbonate, NaHCO_3_. Gallic acid was used as the standard in the experiment.

 A standard curve was constructed using the equation *y* = 0.0026*x* + 0.0402, where *y* is absorbance and *x* is gallic acid content in **μ**g/mL. This equation was used for each extract to determine their percentage of gallic acid content. The total phenolic contents of crude extracts were expressed as gallic acid equivalent (**μ**g of gallic acid/mg of crude extracts).

An aliquot of 100 **μ**L of each solution with different concentrations of gallic acid was added into each well together with 750 **μ**L of FC reagent (previously diluted 10fold with distilled water). A blank control solution (EtOH without extracts) was prepared in the same plate. Consequently, 750 **μ**L of 60 g/L (60 g in 1 L distilled water) of NaHCO_3_ was added into each well and left in the dark for 90 minutes. The absorbance of each extracts was measured at 725 nm. The experiment was executed in triplicate for accuracy. All the previous steps were repeated by different extracts with only one concentration which was 500 **μ**g/mL.

### 2.6. Antimicrobial Assay

 Nutrient agar (NA) and Mueller Hinton agar (MHA) were prepared according to the manufacturer's instructions. The medium was cooled to 50°C after autoclaving, before approximately 25.0 to 30.0 mL of the medium was poured into 15 × 100 mm plastic petri dishes at a uniform depth of 4 mm. Each type of microorganism (from the stock culture) was streaked onto a noninhibitory NA plate to obtain isolated colonies. After an overnight incubation (16–20 hours) at 37°C, one single colony was selected and inoculated with a loop, transferred into 10.0 mL of sterile nutrient broth (NB), and incubated in a shaking incubator at 37°C for 16–20 hours. The density of bacteria was standardized using McFarland 0.5 turbidity standard to 1 × 10^8^ coliform units (cfu)/mL by diluting each suspension in 10 mL of sterile distilled water to the appropriate density.

The antibacterial assay was performed using disc diffusion (Kirby-Bauer) method as described by Aksoy et al. The standardized bacterial suspensions were swabbed on MHA surface using sterile cotton wool. For initial screening, 1 mg of DCM : MeOH extract was loaded onto each Whatman No. 1 filter paper discs (ø, 6 mm) and were impregnated. The positive (streptomycin 10 **μ**g or vancomycin 30 **μ**g) and negative controls (blank disc impregnated with methanol) were used in the antibacterial test. The plates were left at 4°C for an hour to allow for the diffusion of the extracts before they were incubated for 16–20 hours at 37°C, and the diameter of the inhibition zones was then measured [25].

## 3. Results and Discussion

According to the National Cancer Institute (NCI), a crude extract can be considered active if it possesses an IC_50_ value of less than 20 **μ**g/mL [20, 26, 27]. From this screening, Raji was found to have the lowest susceptibility cancer cells among the investigated cell lines. However, the methanol extract of *M. beccariana* demonstrated potent cytotoxic activity towards the Raji cells with an IC_50_ value of 0.98 **μ**g/mL. The hexane extract of *M. beccariana *exhibited moderate to strong activity towards proliferation of the SNU-1, K562, SK-MEL-28, IMR-32, Hep-G2, and NCI-H23. The dichloromethane extract gave a similar result except for a weak dose-dependent concentration against the SK-MEL-28 and Hep-G2 cells. The ethyl acetate extract of *M. beccariana* illustrated moderate cytotoxicity against SK-MEL-28 cells only.

On the other hand, the hexane and dichloromethane extracts of *M. ferrea* displayed moderate to strong antiproliferative effects against all the cancer cell lines except for the Raji cells. The IC_50_ values were in the range of 8.85 to 43.75 **μ**g/mL. However, the DCM extract barely gave any activity towards the HeLa cells, while the ethyl acetate and methanol extract, were inactive towards the proliferation of the tested cell lines.

Additionally, the hexane extract of *Mesua congestiflora *indicated mild cytotoxicities towards SNU-1, K562, and NCI-H23 cells and weak cytotoxic activity towards the rest of the cell lines. Both the ethyl acetate and methanol extracts were not cytotoxic towards all the investigated cells.

**Figure 1:**
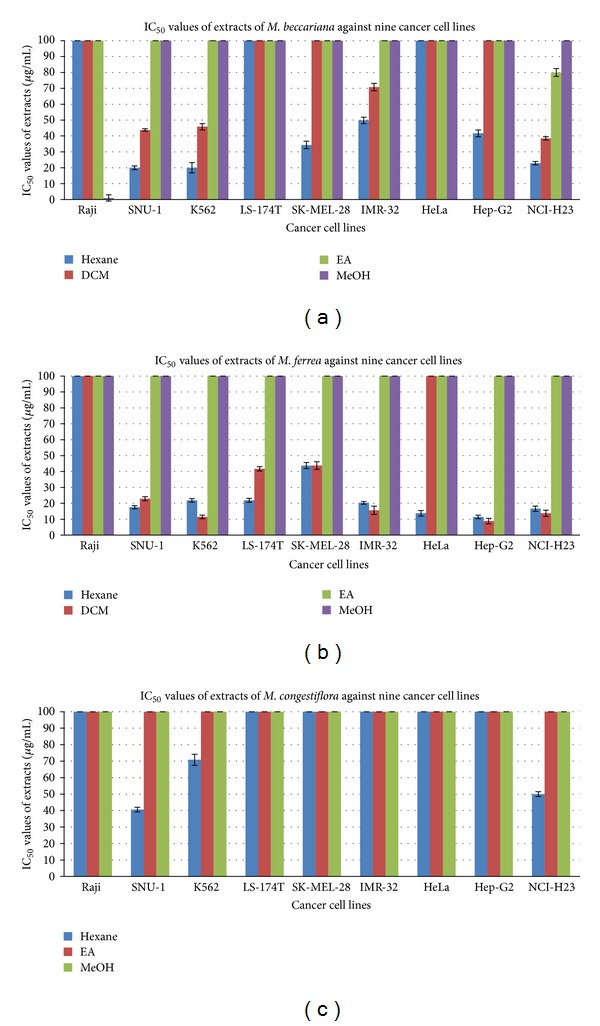
IC_50_ values of the extracts of (a) *M. beccariana*, (b) *M. ferrea, *and (c) *M. congestiflora *against nine cancer cell lines.

In conclusion, only nonpolar to semipolar extracts contribute to the cytotoxic effects on the panel of human cancer cell lines except for the polar extract of *Mesua beccariana *as shown in Table 1 (Figure 1). Quercetin, which was used as a standard, showed cytotoxic activity with IC_50_ values ranging from 2.00 to 31.25 **μ**g/mL. In our previous paper, we reported eleven xanthones from the three plant species, and three of which were very cytotoxic towards all the nine cancer cell lines tested. The structure-activity relationship (SAR) study revealed that the diprenyl, dipyrano, and prenylated pyrano substituent groups of the xanthone derivatives contributed towards the cytotoxicities [22]. Moreover, preliminary tests of cytotoxicity on other isolated metabolites were also reported [9, 21].

All the extracts of *Mesua beccariana*, *Mesua ferrea*, and *Mesua congestiflora *along with ascorbic acid (standard drug) at different concentrations were evaluated for their scavenging potentials on the DPPH free radical.

Several semipolar to polar extracts possess medium to strong scavenging activity against DPPH radical where the methanol (MeOH) extracts of *Mesua beccariana *and *Mesua ferrea *exhibited significant scavenging activity with EC_50_ values of 12.70 and 9.77 **μ**g/mL which are comparable with ascorbic acid (EC_50_ = 5.62 **μ**g/mL). The ethyl acetate (EA) extract of *Mesua beccariana *and *Mesua congestiflora *as well as the dichloromethane (DCM) extract of *Mesua ferrea *reflected mild scavenging capability, while the dichloromethane extract of *Mesua beccariana *gave weak scavenging effect. Besides this, the hexane extract of all three *Mesua *species, the EA extract of *Mesua ferrea, *and MeOH extract of *Mesua congestiflora *were seen to be inactive towards DPPH scavenging agent.

**Figure 2:**
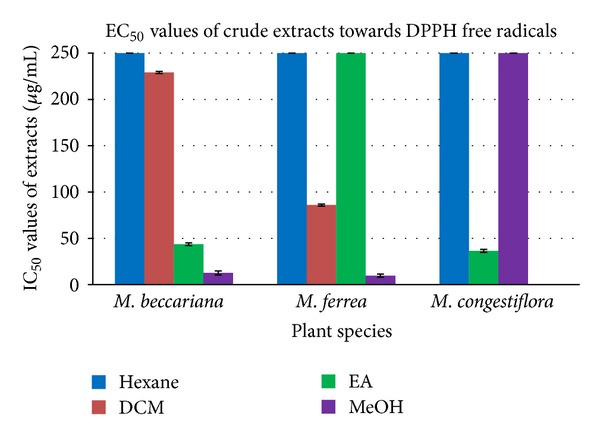
EC_50_ values of crude extracts of the three *Mesua* species towards DPPH free radicals.

 The EC_50_ values for the extracts of three *Mesua* species towards DPPH free radicals are summarized in Table 2 (Figure 2).

**Figure 3:**
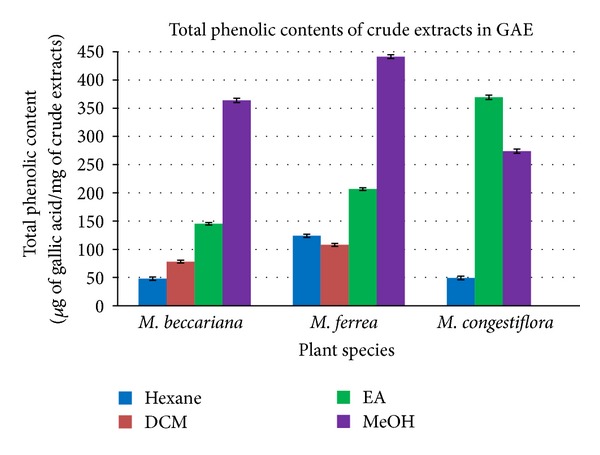
Total phenolic content of crude extracts of the three *Mesua *species.

The total phenolic content (TPC) analysis was carried out on all the crude extracts by using Folin-Ciocalteu method. The TPC values give information on the phenolic contents of the crude extracts and their antioxidant activities. The methanolic extracts of *Mesua beccariana *and *Mesua ferrea *exhibited the highest phenolic contents among the extracts with values of 363.82 and 441.33 **μ**g in gallic acid equivalent (GAE), whereas the ethyl acetate extracts of both species also possess moderate phenolic contents with values of 145.26 and 206.67 **μ**g in GAE. Inversely, the ethyl acetate extract of *Mesua congestiflora *gave a higher phenolic content compared to its methanol extract with values of 369.26 and 273.87 **μ**g in GAE, respectively. The hexane and dichloromethane extracts of the three *Mesua *species contributed low to moderate phenolic contents with GAE values less than 130 **μ**g of gallic acid per mg of extract.  Table 3 summarizes the total phenolic contents of crude extracts in GAE (Figure 3).

The extracts of *Mesua beccariana *were screened against both Gram positive and Gram negative bacteria. Streptomycin and vancomycin were used as positive control standards in the assay. The four types of Gram positive bacteria used were* Bacillus cereus*, *Micrococcus luteus*, methicillin-sensitive *Staphylococcus aureus *(MSSA), and methicillin-resistant *Staphylococcus aureus *(MRSA), while four types of Gram negative bacteria including *Pseudomonas aeruginosa*, *Klebsiella pneumoniae*, *Escherichia coli*, and *Enterobacter aerogenes* were tested in this experiment. All the crude extracts showed negative results in the inhibition of Gram negative bacteria. The methanol extracts, however, showed some activity against *B. cereus*, MSSA, and MRSA. In addition, the hexane extract also gave weak activity against *B. cereus *bacterium.  Table 4 summarizes the antibacterial activity of the crude extracts of *Mesua beccariana*.

## 4. Conclusion

Both DPPH radical assay and total phenolic content indicate that semipolar to polar extracts had high antioxidant properties, while non-polar extracts exhibited good cytotoxic activity. The pharmacognosy investigation showed adverse effects of crude extracts suggesting that the three *Mesua *species could be a good source for the development of lead compounds.

## Figures and Tables

**Table 1:** IC_50 _values of a panel of human cancer cell lines treated with extracts of the three *Mesua *species.

Crude Extracts	Cell lines with IC_50_ values (**µ**g/mL)								
	Raji	SNU-1	K562	LS-174T	SK-MEL-28	IMR-32	HeLa	Hep-G2	NCI-H23
*M. beccariana *									
Hexane	—	20.00 ± 1.15	20.00 ± 3.27	—	34.37 ± 2.32	49.80 ± 2.04	—	41.67 ± 2.18	22.90 ± 1.12
DCM	—	43.75 ± 0.78	45.83 ± 2.00	—	—	70.80 ± 2.36	—	—	38.50 ± 1.08
EA	—	—	—	—	—	—	—	—	80.00 ± 2.43
MeOH	0.98 ± 1.96	—	—	—	—	—	—	—	—
*M. ferrea *									
Hexane	—	17.50 ± 1.02	21.88 ± 1.09	21.88 ± 1.27	43.75 ± 1.91	20.30 ± 0.84	13.75 ± 1.77	11.45 ± 1.09	16.67 ± 1.65
DCM	—	22.91 ± 1.25	11.45 ± 1.09	41.67 ± 1.38	43.75 ± 2.38	15.64 ± 2.62	—	8.85 ± 1.71	13.75 ± 1.92
EA	—	—	—	—	—	—	—	—	—
MeOH	—	—	—	—	—	—	—	—	—
*M. congestiflora *									
Hexane	—	40.63 ± 1.45	70.83 ± 3.37	—	—	—	—	—	50.00 ± 1.44
EA	—	—	—	—	—	—	—	—	—
MeOH	—	—	—	—	—	—	—	—	—

Quercetin	2.08 ± 0.80	6.30 ± 1.93	9.89 ± 3.20	—	21.88 ± 2.07	31.25 ± 2.11	8.00 ± 1.74	5.21 ± 1.85	17.50 ± 1.88

*IC_50 _values more than 100 **µ**g/mL indicate weak activity with symbol (—).

**Each data represents the mean of three independent experiments.

**Table 2:** EC_50 _values of crude extracts towards DPPH free radicals.

Compounds		EC_50 _values (**µ**g/mL)
Crude extracts	*M. beccariana *	
	Hexane	—
	Dichloromethane	229.17 ± 1.22
	Ethyl acetate	43.75 ± 1.43
	Methanol	12.70 ± 2.11
	*M. ferrea *	
	Hexane	—
	Dichloromethane	85.93 ± 1.11
	Ethyl acetate	—
	Methanol	9.77 ± 1.67
	*M. congestiflora *	
	Hexane	—
	Ethyl acetate	36.46 ± 1.56
	Methanol	—

Standard drug	Ascorbic acid	5.62 ± 1.23

*EC_50 _values more than 250 **µ**g/mL indicate weak activity with symbol (—).

**Each data represents the mean of three independent experiments.

**Table 3:** Total phenolic contents of crude extracts in GAE.

Plant species	Crude extracts	Total phenolic content (**µ**g of gallic acid/mg of crude extracts)
*Mesua beccariana *	Hexane	47.95 ± 3.12
	Dichloromethane	78.10 ± 2.45
	Ethyl acetate	145.26 ± 2.23
	Methanol	363.82 ± 3.78

*Mesua ferrea *	Hexane	123.85 ± 2.87
	Dichloromethane	108.05 ± 2.56
	Ethyl acetate	206.67 ± 2.53
	Methanol	441.33 ± 3.33

*Mesua congestiflora *	Hexane	49.21 ± 3.23
	Ethyl acetate	369.26 ± 3.90
	Methanol	273.87 ± 3.77

*Each data represents the mean of three independent experiments.

**Table 4:** Antibacterial activity of crude extracts from *Mesua beccariana* on Gram positive bacteria.

Crude extract (1 mg)	1	2	3	4
Hex	8.33	—	—	—
DCM	—	—	—	—
EA	—	—	—	—
MeOH	7.00	—	8.00	8.67
Streptomycin (10 *µ*g)	18.70	23.00	17.0	not tested
Vancomycin (30 *µ*g)	not tested	not tested	not tested	21.00

*Each data represents the mean of three independent experiments. Symbol “—” represents no activity.

**Each value represents the inhibition zone (mm).

***1-*Bacillus cereus*; 2-*Micrococcus luteus*; 3-methicillin-sensitive *Staphylococcus aureus *(MSSA); 4-methicillin-resistant* Staphylococcus  aureus *(MRSA).

**Table 5:** Weights of the extracts obtained from the three *Mesua* species.

	*M. beccariana* (3 kg)	*M. ferrea* (3 kg)	*M. congestiflora* (0.84 kg)
Hex (g)	15.6	49.6	5.5
DCM (g)	21.2	19.5	—
EA (g)	15.8	16.7	61.0
MeOH (g)	80.5	62.2	120.5
